# Multi-Dimensional Feature Fusion Network for No-Reference Quality Assessment of In-the-Wild Videos

**DOI:** 10.3390/s21165322

**Published:** 2021-08-06

**Authors:** Jiu Jiang, Xianpei Wang, Bowen Li, Meng Tian, Hongtai Yao

**Affiliations:** Electronic Information School, Wuhan University, Wuhan 430072, China; jiangjiu@whu.edu.cn (J.J.); bornlee@whu.edu.cn (B.L.); mengtian@whu.edu.cn (M.T.); hongtaiyao@whu.edu.cn (H.Y.)

**Keywords:** video quality assessment, multidimensional features, convolutional neural network, recurrent neural networks

## Abstract

Over the past few decades, video quality assessment (VQA) has become a valuable research field. The perception of in-the-wild video quality without reference is mainly challenged by hybrid distortions with dynamic variations and the movement of the content. In order to address this barrier, we propose a no-reference video quality assessment (NR-VQA) method that adds the enhanced awareness of dynamic information to the perception of static objects. Specifically, we use convolutional networks with different dimensions to extract low-level static-dynamic fusion features for video clips and subsequently implement alignment, followed by a temporal memory module consisting of recurrent neural networks branches and fully connected (FC) branches to construct feature associations in a time series. Meanwhile, in order to simulate human visual habits, we built a parametric adaptive network structure to obtain the final score. We further validated the proposed method on four datasets (CVD2014, KoNViD-1k, LIVE-Qualcomm, and LIVE-VQC) to test the generalization ability. Extensive experiments have demonstrated that the proposed method not only outperforms other NR-VQA methods in terms of overall performance of mixed datasets but also achieves competitive performance in individual datasets compared to the existing state-of-the-art methods.

## 1. Introduction

With the recent popularity of smart devices, video-based application services have become popular, resulting in an increase in the demand for high-quality video. Therefore, the accurate perception of video quality is of essential importance for video sharing and streaming platforms. As the foundation of inpainting and enhancement of low-quality videos, video quality assessment (VQA) methods have become a promising research field in the past few decades. Quality evaluation methods contain two categories: subjective ones and objective ones. In general, subjective video quality evaluation by human experts is considered to be the most accurate method [1]. The scores obtained from subjective methods are often taken as the ground truth of objective evaluation during the training process [2]. However, this approach is quite time-consuming and expensive, which renders it impractical to test a large number of videos transmitted in real-time. By contrast, objective quality assessment by a computer is cheap and efficient. Objective VQA methods can mimic the human visual system (HVS), distinguish distortion in the video, and thus reasonably perceive the quality of the video. Depending on the availability of reference videos, VQA methods contain three categories: full reference (FR) [3,4,5,6], reduced-reference (RR) [7,8], and no-reference (NR) [9,10]. Since videos without distortions can be used as the evaluation criterion, in most cases the video scoring results of the FR/RR methods can achieve satisfactory similarity to the results of human perception. When evaluating the quality of videos captured in real-time, however, it is difficult to obtain distortion-free videos as references. Thus the importance of NR-VQA methods can never be overemphasized.

Among the existing studies, one category of NR-VQA methods perceives artificial distortions generated in the laboratory, while the other category perceives real distortions. Current NR-VQA methods for synthetically distorted videos have achieved phenomenal success. Nevertheless, a significant proportion of videos in real life are obtained by shooting in the wild. Hence, NR-VQA metrics for authentically distorted videos have become a matter of concern. Compared to videos with manual distortions, the frames of the in-the-wild videos contain various types of objects, some of which are beyond the cognitive scope of existing models. Meanwhile, due to camera movement, abnormal exposure, and being out of focus, distortions are randomly distributed in the video, causing difficulties during the feature extraction process. We believe that the NR-VQA method for authentic distortion currently has two challenges: firstly, the selection of features; and secondly, the formation of temporal memory.

In recent years, the application of deep learning in video processing tasks (e.g., action recognition) has gradually attracted attention. Unlike image processing tasks, video analysis tasks focus not only on the information contained in a single frame but also on the association among frames in a certain period, which are referred to as spatial and temporal information, respectively. Spatial features are usually extracted by using 2D convolutional networks such as AlexNet [11], VGG [12], and ResNet [13], while optical flow networks [14,15,16,17], 3D convolution [18,19,20,21], recurrent neural networks [22,23], etc., are often used to obtain temporal features or associations. These experiences inspire objective VQA. Since research has proven that image quality assessment results are intrinsically associated with the awareness of content [24], we can instinctively assume that there is a correlation between video quality evaluation and motion esthesia.

Based on this intuition, we propose a multi-dimensional hybrid feature network to process static spatial features and dynamic temporal features for NR-VQA. Specifically, spatial features are content-related features formed by 2D convolutional networks, and temporal features include motion-related features between adjacent frames (or a clip) formed by high-dimensional convolutional networks. The characteristics (spatial and temporal) contain a wealth of underlying information. For the purpose of extracting the information from a long-term sequence and modeling temporal memory, we employ a structure containing recurrent neural networks, which is called the temporal memory module (TMM) in this article. In order to better fit the subjective scoring data set, we simulate the time hysteresis effect in human visual habits by using a self-generated parametric network and controlling the impact of historical quality for the overall evaluation accordingly. Apart from these, we use multiple datasets for network training, thereby ensuring that the model has a better generalization performance.

The main contributions of this paper are as follows: (i) unique multi-dimensional spatiotemporal feature extraction and integration strategies for objective NR-VQA; (ii) the design of a time-memory module containing recurrent neural networks for long-term sequence-dependence modeling; and (iii) adaptive parameter networks to imitate the impact of historical quality on assessment score predictions.

The remainder of the paper is organized as follows. In Section 2, previous works on NR-I/VQA are reviewed, while Section 3 contains the introduction of the proposed approach in detail. Section 4 exhibits the experimental validation of the method and related techniques on mainstream VQA databases with corresponding analysis. Finally, the paper is concluded in Section 5 with possible future directions for research in this area.

## 2. Related Works

In this section, we provide a brief summary of the existing related methods for NR I/VQA tasks. Similar to the image quality evaluation metrics based on classic methods [25,26,27,28,29] and deep learning [30,31,32,33,34], most of the VQA methods can be divided into two steps: feature extraction and (feature-based) quality assessment. In addition, HVS characteristics, such as the influence of ambient illumination level [35] and temporal hysteresis effect [36], are equally important. The difference is that VQA tends to emphasize more on spatio-temporal motion information. For instance, discrete cosine transform (DCT) domain natural scene statistics (NSS), the motion coherency-related algorithm v-BLIINDS by Saad et al. [37], and features in 3D discrete cosine transform (3D-DCT) domain based on spatiotemporal natural video statistics (NVS) were also proven to be effective [38]. Wu et al. [39] proposed an NR-VQA metric to estimate SSIM for single video frame. For video mean subtracted contrast normalized (MSCN) coefficients and spatiotemporal Gabor bandpass filtered outputs, [40] established an asymmetric generalized Gaussian distribution (AGGD) model to perceive distortions. In the meantime, optical flow [4,41], ST-chip [42], multi-scale trajectory [43], and bitstream level features [44,45,46,47] were also used to quantify distortion in video data. Although many of these methods contribute greatly to the perception of specific distortions without reference, they are not satisfactory for evaluating the quality of in-the-wild videos with sophisticated distortion.

The success of CNN networks in object detection, instance segmentation, video understanding, and other fields has aroused attention in VQA researchers. Specifically, the presence of perceptual similarity [24,48] showed that quality analysis is intrinsically linked to object recognition, and thus it will be effective to use existing pre-trained CNN models for video quality analysis. The authors of [49] provided an efficient deep-learning metric called DIQM to reduce the computational complexity in mimicking the HVS. For perceivable encoding artifacts (PEAs), [50] proposed a CNN network for identifying different kinds of distortions. For convolutional neural networks and multi-regression-based evaluation (COME), [51] proposed a multi-regression model to imitate human psychological perception. Concerning the limitation of HDR-VDP 2, [52] developed NoR-VDPNet to predict global quality with substantially lower computational cost. Wei et al. utilized Semantic Information related two-level network to estimate the image quality [53]. Entropic differences learned by the CNN network were used to capture distortions in [54]. In order to enable the model to have the ability of time-series memory, recurrent neural networks are used in many metrics. For example, Li et al. [55] trained a GRU with CNN features for NR-VQA in order to obtain a perception of video frame content and distortion. The combination of 3D-CNN and LSTM was used in [56] for distortion perception. With the help of transfer learning and temporal pooling, [57] developed a new NR-VQA architecture. In this paper, we construct a GRU-based structure with jump connections for temporal memory. On the one hand, this can solve long sequence dependence; on the other hand, this reduces information loss.

For NR-I/VQA, there are already some databases suitable for training deep learning networks, such as UPIQ [58], CVD2014 [59], KoNViD-1k [60], LIVE-Qualcomm [61], LIVE-VQC [62], etc. Network models trained for a specific database often perform poorly in terms of prediction in other databases due to the differences between individual databases. In order to help the models in obtaining better generalization performance, researchers have recently proposed some methods for cross-dataset training. Zhang et al. [63] built a training set with image pairs in order to avoid subjective quality evaluation for different datasets. Based on the study of different feature distributions for different datasets, UGC-VQA [64] considered a selected fusion of BVQA models to reduce the inconsistency in subjective assessment among datasets. Li et al. [65] divided the evaluation of network prediction scores into three steps: relative quality esthesia, perceptual quality awareness, and subjective quality generation, followed by a multi-parameter structure for the transitions between tiers to suit different datasets.

As described in this section, the fusion of multi-dimensional CNN features, which was proven to be quite effective in video understanding, has rarely been taken into account in the VQA task. Therefore, exploring appropriate fusion methods and designing reasonable processing frameworks for them in time series can be considered a promising area of research.

## 3. Proposed Method

**Figure 1:**
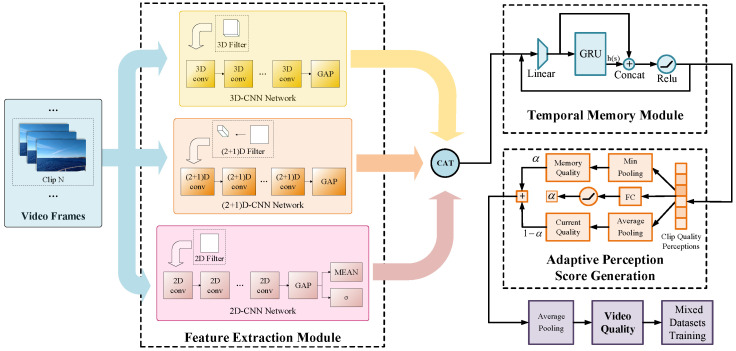
A visual illustration of our proposed multi-dimensional feature fusion network: feature extraction module, temporal memory module, and adaptive perception score generation module.

In the proposed method, a fusion of multidimensional CNN features is considered. Such features are very common in video understanding tasks such as action recognition but are rarely used in VQA tasks. As shown in Figure 1, our network structure can be divided into three parts: (i) a multidimensional feature fusion module, (ii) a temporal memory module, and (iii) an adaptive perception score generation module. In the multidimensional feature fusion module, different network structures and convolutional kernels are utilized to process the video sequences and, thus, generating rich spatio-temporal features. We then fuse these features and place them into the temporal memory module. In the temporal memory module, in order to generate features containing previous temporal memory information, we use a special recurrent network structure with shortcuts to form evaluation memories over the entire video sequences. In the score generation module, we use a self-generated parameter structure for coping with the impact of image frame quality variations on overall quality perception.

### 3.1. Multidimensional Features Fusion

**Figure 2:**
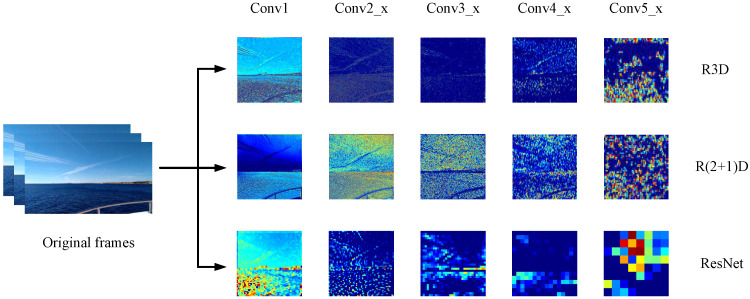
Feature maps generated by convolutional networks with different dimensions.

The purpose of multi-dimensional fusion is to obtain rich features that are subsequently propagated to the network for characterizing spatio-temporal information by using different convolutional kernels/different network structures when processing original video clips. The feature maps generated by convolutional networks with different dimensions are shown in Figure 2. We used ResNet networks [13], R(2+1)D networks, and R3D networks [21], which have similar structures. In addition to 2D-CNN, we select two different multi-dimensional features because, on the one hand, their temporal and spatial meanings are different; on the other hand, it is necessary to prevent spatiotemporal information imbalance.

In the image/video quality evaluation task, subjective evaluation results are shown to be correlated with content such as scenes and objects [55]. Advances in deep 2D convolutional neural networks in fields such as object recognition suggests that 2D-CNN can competently mimic the human perception of static content in video sequences. Simultaneously, the deep features generated by such networks have been proved to be distortion-sensitive [66]. Therefore, 2D-CNN backbones for image recognition, such as ResNet, are regularly used in image/video quality assessment. Typically, these networks are initially pre-trained on image classification databases such as ImageNet to generate feature maps related to static content/distortion. Evaluation scores are then obtained by using the deep feature maps after subsequent processing. In addition, it is fairly common to generate evaluations by using transfer learning. As for video tasks, in parallel to static scenes and objects, human perceptual content also consists of temporally manifested motion. When imitating the human visual system, in addition to focusing on 2D scene/object features, a 3D convolutional network can be used to pay attention to the effect of low-level motion content on the evaluation results.

Suppose the video *V* contains *n* image frames $I_{i}\left(i=1,2,\ldots ,n\right)$, each adjacent *t* frames form a clip (generally, t=3). Then, there are *m* image clips $Cli_{j}\left(j=1,2,\ldots ,m\right)$, where m=n/t. Then the features extracted by CNN models of different dimensions can be denoted as follows(FD denotes 2D-CNN features, FP denotes (2+1)D-CNN features and FT denotes 3D-CNN features):

(1) $$FD_{i}=2DCNN\left(I_{i}\right),$$

The 2D-CNN convolves individual image frames, while 3D-CNN convolves a clip of several image frames.

(2) $$FP_{j}=\left(2+1\right)DCNN\left(Cli_{j}\right),$$

(3) $$FT_{j}=3DCNN\left(Cli_{j}\right).$$

After the convolution operation is the Global Average Pooling (GAP) layer, which transforms the feature maps FD, FP, and FT into feature vectors fd, fp, and ft, thus, enabling the recurrent neural network to be used for memorization. In order to avoid excessive information loss from GAP operations on 2D convolutional features, we also use global standard deviation pooling (GSP) to obtain variation information. Finally, the outputs of two pooling layers are concatenated as follows.

(4) $$fp_{j}=GAP\left(FP_{j}\right),$$

(5) $$ft_{j}=GAP\left(Ft_{j}\right).$$

(6) $$fd_{i}=GAP\left(FD_{i}\right)⊕GSP\left(FD_{i}\right),$$

The concatenation operation is denoted by ⊕.

**Figure 3:**
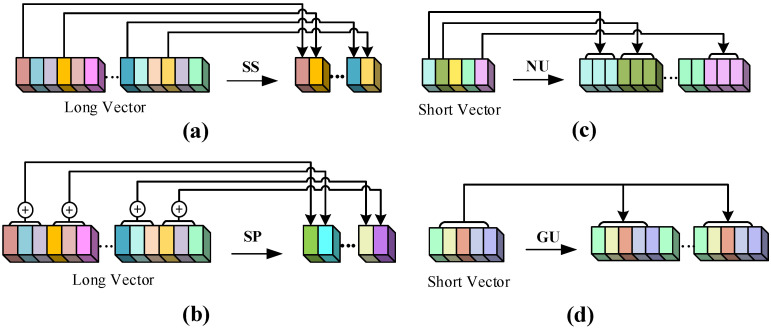
Feature vector size alignment. (**a**) Long vector sub-sampling (SS). (**b**) Long vector sumpooling (SP). (**c**) Short vector nearest-neighbors upsampling (NU). (**d**) Short vector global upsampling (GU).

For each frame, the network generates a 2D convolutional feature vector; however, for *t* frames, there is only one 3D feature vector and one (2+1)D feature vector, which results in the feature-length difference in time sequence. As shown in Figure 3, in order to align vectors for the concatenation operation, we consider two kinds of rescaling methods: shortening the long vector and amplifying the short vector. Shortening methods include long vector sub-sampling and sum-pooling, while amplification methods include nearest-neighbors upsampling and global upsampling.

The long vector sub-sampling means selecting only the vector generated in one of the *t* image frames as the representative vector for the concatenation operation. In sumpooling, we sum the *t* features in a clip to avoid information attenuation caused by subsampling. The nearest-neighbors upsampling alignment is implemented by replicating *t* times for each clip-generated feature, and the global upsampling alignment copies the feature vector of all clips in a video *t* times to make the long and short vectors the same length. In subsequent experiments, we find that long vector sub-sampling loses a large amount of two-dimensional perceptual information, resulting in poor model performance, while the global upsampling method performs relatively better. Let the vector length be *L*; after aligning the three vectors, we perform a concatenation operation on them to obtain the features *f* containing spatio-temporal information.

(7) $$f=f_{d}⊕\underset{L=n}{GU}\left(f_{p}\right)⊕\underset{L=n}{GU}\left(f_{t}\right).$$

### 3.2. Temporal Memory Module

**Figure 4:**
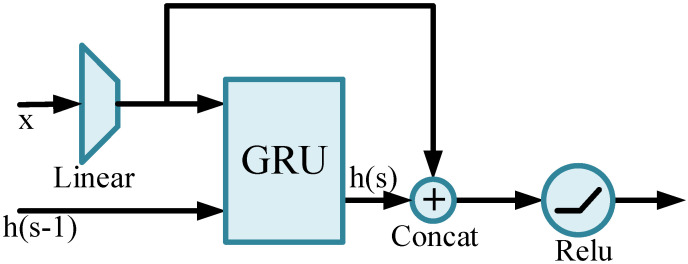
Temporal memory block consists of a GRU branch and a linear branch.

In the above subsection, we use 3D-CNN to model the connection between adjacent frames. The feature *f* can be considered as an encoding of low-level motion characteristics. In order to further develop a long-time series modeling for high-level features, we use recurrent neural networks (RNN). GRU [23] is one of the typical recurrent neural networks that use a gating mechanism to control input, memory, and other information to make predictions based on the current time step. It has the advantage of preserving information in long-term sequences and will not remove it even if it is not correlated with the prediction results. In the network, we implement a multi-level cascade of fully connected layers and recurrent networks. On top of GRU, we add a short path to enhance the learning ability of the network, the structure of which is depicted in Figure 4. This temporal memory block (TMB) is composed of a GRU branch and a shortcut branch, representing historical quality memory and current quality perception, respectively, and thus avoiding an excessive loss of information. Specifically, we concatenate the GRU hidden state $h\left(s\right)$ at the current moment *s* with the input information *x* after dimension reduction and finally feed the result into the nonlinear activation layer.

In order to enhance the understanding of the time series information, we link several TMB blocks and then use a fully connected layer to generate a history-related quality score for each clip. The scores of all clips in a video form a video rating vector $q_{c}$.

### 3.3. Adaptive Perception Score Generation

**Figure 5:**
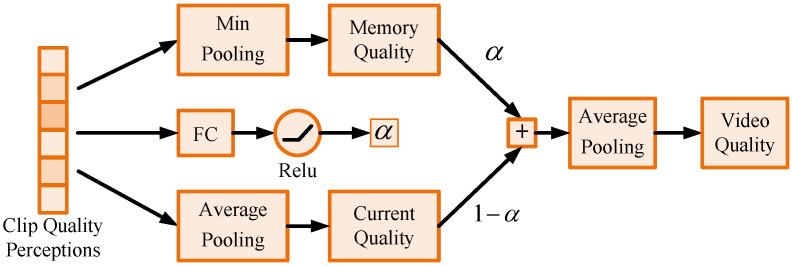
Adaptive Perception Score Generation. Parameter α is generated by clip-level quality perception scores with a fully connected layer and a nonlinear layer.

By processing all clips in the video, we generate an array of scores associated with the historical quality impact. In this subsection, we introduce a parameter-adaptive video score generation strategy. Research has shown that a decrease in video quality compared to an enhancement results in the scorer being more impressed. This phenomenon is referred to as the temporal hysteresis effect [36]. It can be inferred that when there are poor quality clips in the video sequence, the rating perception will drop significantly, while it is not so sensitive to rising quality. Based on this, we try to generate video quality scores *Q* using clip scores $q_{c}$ (see Figure 5). As mentioned in [55], the final evaluation score consists of two components: memory of the historical worst perceptions $Q_{m}$ and the current rating status $Q_{c}$.

(8) $$Q_{m}=\min\left(q_{c}\right),$$

(9) $$Q_{c}=\sum_{m\in q_{c}}mw,$$

$Q_{m}$ is generated by a Min pooling block, $Q_{c}$ is generated by a Softmin-weighted average pooling, *w* is a parameter generated by a differentiable Softmin function. Subjective frame quality scores can be approximated by linearly combining the $Q_{m}$ and $Q_{c}$ with parameter α, as follows.

(10) $$q_{c}^{\prime }=\alpha Q_{m}+\left(1-\alpha \right)Q_{c}.$$

In the human visual system, the memory of history is also affected by the current status. If the current clip has exceptional performance (relatively good or relatively poor), the test subjects will be impressed, while if the current frame performance is relatively mediocre, the test subject will recall the previous scenes more often. Thus, the proportion of these two components in the final evaluation system should be dynamic. Therefore, we design an adaptive weight α generation structure using an FC layer and a nonlinear activation layer.

(11) $$\alpha =FC(Relu\left(q_{c}\right)).$$

During the training process, the network can learn the weight α on its own with the help of the score vector $q_{c}$.

### 3.4. Implementation Details

In this paper, we use ResNet50 pre-trained on the ImageNet [67] dataset and later fine tuned the image quality evaluation task [68] as a 2D-CNN feature extractor for a better perception of the distortions. We extract 2D features from the ‘res5c’ layer in ResNet50 and then set the feature size to 4096 after pooling. R(2+1)D-18 and R3D-18 [21], which are pre-trained on the human action recognition dataset Kinetics [69] from the ‘conv5_x’ layer, are chosen for multidimensional feature extraction, which provides the ability to perceive motion information. After the pooling operation, the feature sizes are both 512. The feature extraction module is separated from the model training process in order to avoid excessive computing time consumption. In order to form temporal memory, we consider third-order TMB blocks. The dimensions of each block are shown in Table 1. The learning rate in our work is set to $1\times 10^{-4}$ and Adam is used as the optimizer to train our model for 40 epochs, with a batch size of 32. As in [65], the model loss is defined as the softmax weighted average of the numerical summation of L1 loss, monotonicity-related loss, and accuracy-related loss in each dataset. We implement our model using PyTorch and conduct training as well as testing on a single NVIDIA 1080Ti GPU.

## 4. Experiments and Discussion

In this section, we present a study of NR-VQA with the framework mentioned in the previous section on four benchmark datasets, as they are all authentically distorted. We introduce four benchmark databases with mixed distortions in detail firstly. Then, we conduct a performance comparison and result analysis on our method and several popular NR-VQA models. After that, ablation experiments and temporal strategies experiments are conducted.

### 4.1. Experimental Setups

#### 4.1.1. Experimental Datasets

In order to improve the generalization ability and performance of the model, we train and validate the model on four public authentically distorted video datasets: Camera Video Database (CVD2014) [59], Konstanz Natural Video Database (KoNViD-1k) [60], LIVE-Qualcomm [61], and LIVE Video Quality Challenge (LIVE-VQC) [62]. The main characteristics of these four datasets are summarized in Table 2.

- CVD2014 [59] database consists of 234 videos recorded by real cameras, which results in distortion complexity. For every distorted video, there is a mean opinion score in the range [−6.50,93.38]. The resolution of the video in the database includes both 640×480 and 1280×720.
- KoNViD-1k [60] database is the largest in terms of video volume among these four, containing 1200 videos with diversity in terms of semantics, context, and types of visual distortions. In addition to scenes shot directly with a camera, video samples of KoNViD-1k also include other content such as animation and time-lapse photography.
- LIVE-Qualcomm [61] mainly focuses on video content generated by users. What renders it different from other databases is that LIVE-Qualcomm only provides full HD videos of resolution 1920×1080 shot by several mobile phones. Six types of in-capture distortions are modeled in LIVE-Qualcomm: artifacts, color, exposure, focus, sharpness, and stabilization.
- LIVE-VQC [62] includes 585 videos of unique content with a wide range of complex and authentic distortions. Videos in this database are captured by mobile camera users without restrictions on content or capture style. Moreover, in order to collect a large number of MOS, thousands of participants took part in the assessment task, generating over 205,000 opinion scores by crowdsourcing.

#### 4.1.2. Evaluation Metrics

Similar to image quality evaluation, Spearman’s rankordered correlation coefficient (SROCC) and Pearson’s correlation coefficient (PLCC) between the predicted and the ground truth scores are commonly calculated as the evaluation criteria in VQA. These two indices, ranging from −1 to 1, provide a good representation of the prediction monotonicity and accuracy. The larger the indices, the better the performance. During the training and testing stages, we randomly split these four datasets into independent training and testing sets: 80% is set for training, and 20% is set for testing. Moreover, 25% of the training data is used for validation. We repeat experiments on split data 10 times, and the mean indices are given as the algorithm performance evaluation.

### 4.2. Experimental Results and Comparisons

#### 4.2.1. Single Database Evaluations

First of all, we test the cross-dataset adaptability of the method. We present all metric results of training on a single dataset and testing on another single dataset. The average SROCC scores are listed in Table 3. Of the nine combinations of training and test datasets, our method outperforms the existing state-of-the-art methods in seven cases, performing second in the rest. In particular, our method delivers favorable properties when the test sets are CVD2014, KoNViD-1K, and LIVE-VQC. The superior performance proves that our model is highly adaptable to training across datasets, which, on the other hand, justifies exploring the internal links between video quality evaluation datasets by fusing the features of different dimensions and using different temporal memory structures.

**Figure 6:**
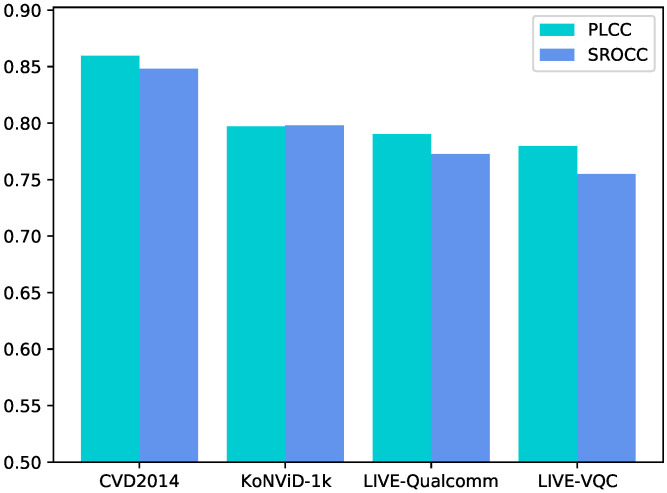
SROCC/PLCC performance on single video database.

We proceed to present a study of no-reference video quality assessment performance on a single training/testing database for the multidimensional feature fusion method presented in the previous section. Table 2 reports the metric comparison of our method with other methods. Instinctively, the model behaves best when the train set and the test set are sourced from the same video database. Performances on a single database training/testing process are given in Figure 6, which indicates that our network has a fairly good perception of video quality.

#### 4.2.2. Generalization Ability

In order to evaluate the effectiveness of the proposed model, we first analyze the general performance of our model with five prominent NR-VQA models (BRISQUE [26], VIIDEO [72], VBLIINDS [37], TLVQM [2], and MDTVSFA [65]) on CVD2014, KoNViD-1k, and LIVE-Qualcomm. All the models are trained with mixed databases, and the indices are calculated by weighted average, with the weight generated by the database size. As shown in Table 4, our approach (the seventh column) demonstrated the best overall performance on three benchmark databases, especially in SROCC metrics, i.e., the proposed multi-dimensional network presents a favorable prediction of monotonicity in NR-VQA.

We further present a performance comparison of our approach on an individual database (see Table 5). It should be mentioned that, with the exception of MDTVSFA and our proposed model, other methods are individually trained on a single database. Among the performances on the four authentically distorted video databases, our method performs favorably versus the state-of-the-art NR-VQA methods. In particular, we have made significant progress on the LIVE-VQC database and CVD2014 database, which validates the effectiveness of our model. The authors of [56,57] outperform our metic on KoNViD-1K, while [56] underperforms ours on LIVE-Qualcomm, and [57] benefits a lot from transfer learning (values in brackets are transfer-learning induced performance increases). Compared to these methods, our method, without transfer learning, performs well for mixed datasets training.

**Figure 7:**
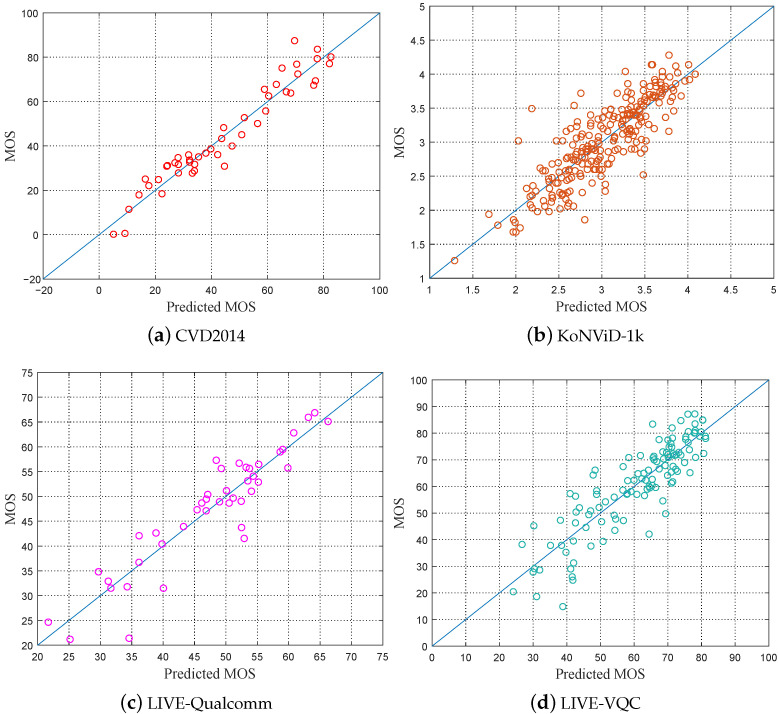
Scatter plots of the subjective scores provided by the dataset versus the objective scores provided by our method.

In addition, Figure 7 presents the scatter plots of the subjective MOSs in four datasets versus the predicted scores of the proposed method. We used the trained model to perform score prediction on the test sets of the four datasets. It can be observed from Figure 7 that, for each test set, the predicted scores and ground-truths are evenly distributed on a diagonal line from the bottom left to the top right, showing a good linear relationship.

**Figure 8:**
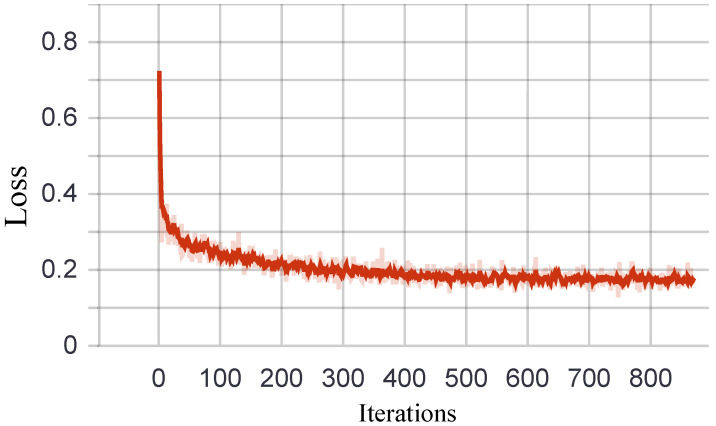
The relationship between loss value and the number of iterations in the training phase.

**Figure 9:**
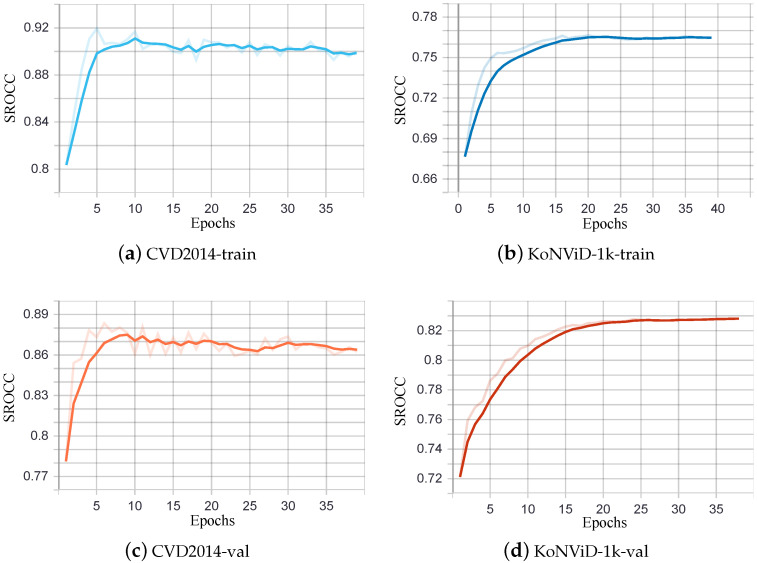
SROCC performance in each dataset over the training and validation epochs.

In order to better visualize the training phase, we report the decrease in loss when the number of iterations increases in Figure 8 and the change of SROCC with the increase in epochs on each dataset in Figure 9.

### 4.3. Ablation Experiments

#### 4.3.1. Integration of Different Dimensions Feature

In order to verify the effectiveness of the fusion features proposed in this paper, we tested the performance of single features and their various combinations on the four datasets (see Table 6).

Our feature extractor includes two categories: content-sensitive ResNet50 and motion-sensitive R3D, R(2+1)D features. From Table 6, we find that dynamic features alone cannot achieve a good perception of video quality. When a certain type of feature is used as an input, content-aware Resnet50 is better than motion-aware features. When mixing two types of features, the performance of the spatio-temporal feature fusion is better than the fusion of the temporal features. Moreover, the combination of the three features performs best. We can conclude that our multi-dimensional feature fusion metric helps the model improve its ability to analyze the dynamic and static distortions present in video clips.

Table 7 compares our concatenate method with another fusion method: sum fusion. Sum fusion calculates the sum of spatio-temporal features over the time series. The result shows that concatenation fusion preserves temporal and spatial information wholly and separately, while the sum method loses the unique nature of characteristics.

#### 4.3.2. Two-Dimensional Feature Extractor Comparison

**Figure 10:**
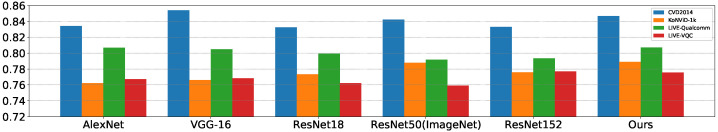
Mean SROCC performance after replacing different 2D feature extractors, including AlexNet, VGG-16, ResNet18, ResNet50 (pre-trained on ImageNet), ResNet152, and ResNet50 finetuned on [68].

In order to verify the effectiveness of our content-aware part, we further used different spatial feature extractors to replace the 2D feature extractor in this paper. We used four data sets for joint training and evaluated the content perception ability of each network by comparing the average SROCC. In particular, we tested the difference in the performance of the Resnet50 model before and after fine-tuning. The result is shown in Figure 10. Our 2D feature extractor performs the best overall. VGG-16 and ResNet152 achieved the best results on CVD2014 and LIVE-VQC, respectively, but performed poorly on the other three datasets. Our model performed the second-best on CVD2014 and LIVE-VQC but obtained the highest mean value on the remaining two datasets. Moreover, the results on all datasets show that our ResNet50 is more suitable for quality perception than the one without fine-tuning.

#### 4.3.3. Evaluation on Long-Term Dependencies Modeling

Establishing long-term dependencies is an equally important part of feature extraction in our framework. In temporal memory modeling, LSTM and GRU usually perform similarly. However, GRU can converge more easily in the training phase because of owning fewer parameters. We further evaluated the effect of using the self-attention mechanism in long-sequence modeling by Transformer [74]. In Table 8, we report the comparison of using GRU, LSTM, and Transformer. It is clear from the table that GRU shows the best long-term modeling ability on most datasets, while LSTM performs slightly worse. In the meantime, the long-term dependency modeling ability of the Transformer is not as good as RNN in our framework.

#### 4.3.4. Evaluation on Adaptive Parameter

**Figure 11:**
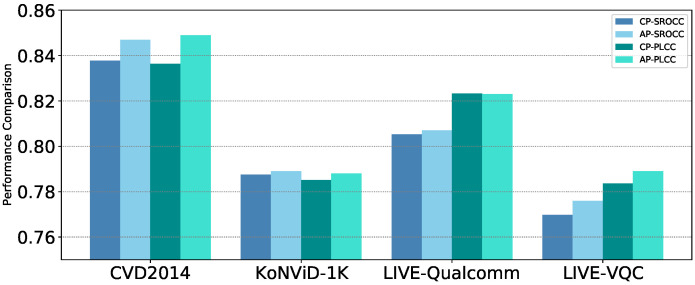
Performance of CP vs. AP on four benchmark databases. Abbreviations CP for constant parameters and AP for adaptive parameters (In CP, α is set to be 0.5).

We also conducted further experiments on four datasets in order to verify the effectiveness of the adaptive parameter α. The average SROCC and PLCC values are given in Figure 11. Relaxing the parameters in order to be trainable allows the model performance to improve on all datasets. Although in [43] α was set to 0.5 as the optimal choice, the results demonstrate that our approach of generating the weight parameter based on the current frame quality results in improved model performance.

### 4.4. Temporal Features Related Strategies

In this subsection, we investigate the temporal alignment of spatio-temporal features and the selection of the number of TMB blocks. We conduct experiments on the KoNViD-1k dataset because it has the largest number of videos.

#### 4.4.1. Feature Alignment Strategy

**Figure 12:**
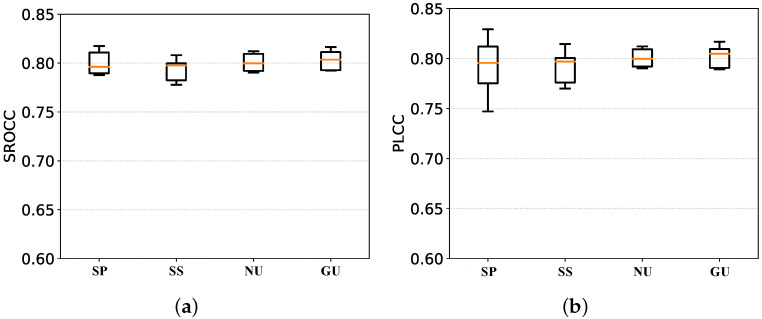
Comparison of box plots for the four alignment methods. (**a**) SROCC of four alignment strategies. (**b**) PLCC of four alignment strategies.

As presented in Section 3.1, 2D features have been generated for each frame in the proposed method, while it takes three frames to generate a 3D feature, which results in a temporal mismatch of features with different dimensions. The experimental results show that the mean SROCC value is 0.785 when the sub-sampling strategy is used and 0.795 after sumpooling; this then rises to 0.796 when the nearest-neighbors upsampling strategy is used and 0.798 with global upsampling is observed. As for PLCC, it is 0.785 when sub-sampling 2D features, 0.792 after sumpooling, 0.795 when nearest-neighbors upsampling, and 0.797 when global upsampling. Box plots of the three strategies’ performance are provided in Figure 12. The two short horizontal lines at the top and bottom of the box plot represent the maximum and minimum values, respectively, and the short horizontal line in the middle represents the median. The shorter the vertical direction is, the larger the median is, which suggests a better model performance. As can be observed from the graph, the global upsampling method performs significantly better than the other three methods in terms of median, while it performs similarly to the nearest-neighbors upsampling method in terms of extreme deviation. The sub-sampling and pooling methods perform relatively poorly, which may be because the sub-sampling method loses too much information, and the pooling method fails to retain the difference and other information. Intuitively, the global upsampling operation does not add new information, but it preserves and enhances short features coherently, which may facilitate the modeling of long-term dependencies.

On balance, the global upsampling method outperforms the other three alignments on the KoNViD-1k database. As a consequence, we chose to upsample the 3D vector so that it has the same width as the 2D features in terms of temporal order in order to perform the concatenation operation.

#### 4.4.2. Choice of Temporal Memory Modules

**Figure 13:**
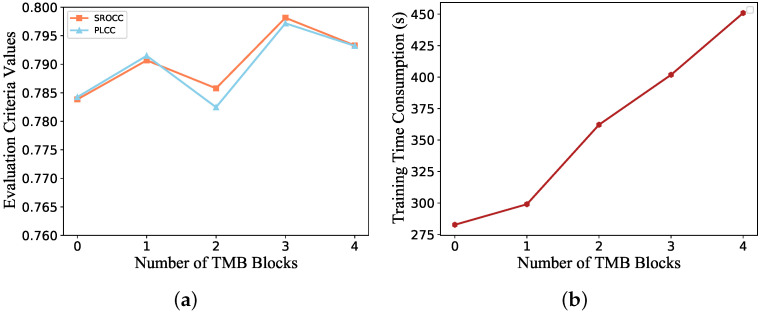
Evaluation of indicators for the selection of TMB. (**a**) Performance indices. (**b**) Training time efficiency on the same database.

In order to explore what kind of temporal memory module is the most appropriate for the fused multidimensional features, we conducted experiments here for different temporal memory modules. Our choice is mainly based on two aspects: perceptual accuracy and the time consumption of the training stage. Figure 13 illustrates the model performance and the time consumption during the training phase when the number of TMBs is changed (the number of 0 means that only a single GRU is used). As can be observed from the graph, the model performance does not increase incrementally as the number increases, but the time consumption rises gradually. One possible reason for the drop in performance at number 2 is that model performance may also be affected by dimensionality of TMB, which results in their values being non-monotonic. There is a small increase in time consumption when changing individual GRU to TMB and a larger increase when adding more TMBs. Considering both the time efficiency and model performance, we chose the number of TMBs to be three.

### 4.5. Computational Complexity

**Figure 14:**
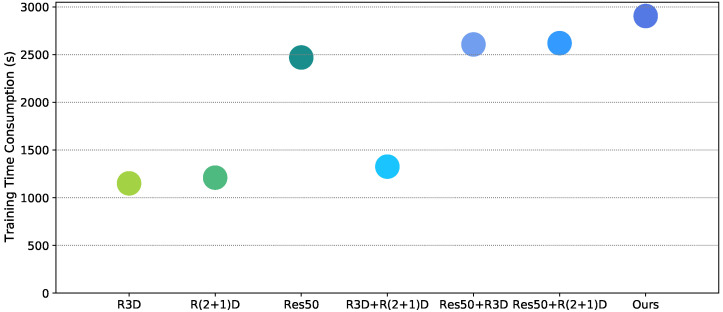
Time consumption in the whole training phase (40 epochs) when using different temporal or spatial features and their combinations.

The increase in time consumption over the whole training phase caused by the number of TMBs has been investigated in Figure 13b. Moreover, changes in training time consumption by using different features are reported in Figure 14. The time spent on the training phase mainly depends on the length of feature vectors. The introduction of multi-dimensional features does not significantly increase the time for mixed datasets training.

**Figure 15:**
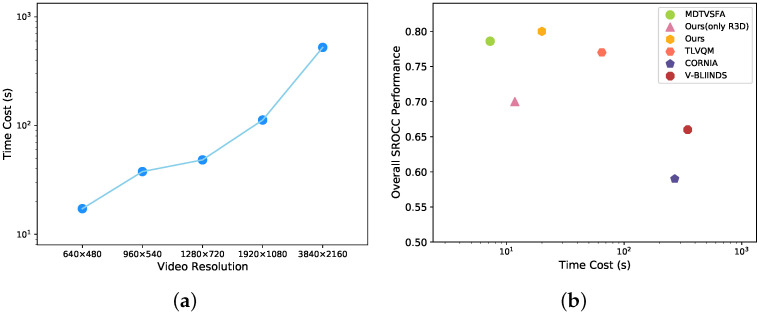
Runtime comparison. (**a**) Time costs in evaluating videos with different resolutions. (**b**) Time costs and overall performance comparison with different metrics.

Finally, the computational complexity of quality-aware algorithms is tested by comparing the time consumption of evaluating videos. All tests were run on a computer with Xeon Gold 5220R CPU, 2x Quadro RTX6000 GPU, and the operating system is Ubuntu 18.04. We first considered the effect of different video resolutions on the evaluation time. The computation time consumed on videos with 300 frames from 480p to 4K can be observed in Figure 15a. For comparison with other metrics, a sample video with a resolution of 640 × 480 and 364 frames was chosen from the CVD2014 dataset for the test. As the results in Figure 15b are observed, the proposed method has the best performance without excessive increase in computational complexity. It is worth mentioning that feature extraction and fusion results in an increase in computation time. The timing module and dynamic parameters, however, have almost no influence on the overall evaluation time because it takes only about 0.02 s in the features processing network.

## 5. Conclusions and Future Work

In this paper, we have presented an empirical study of the temporal effects in objective NR-VQA. Many current approaches are based purely on the content-aware or motion-aware feature while ignoring other equally important features. In addition, finding a better temporal network for perceiving video quality is also worth investigating. With these motivations, we creatively fuse content-oriented 2D-CNNs with motion-oriented 3D-CNNs and complement them with (2+1)D-CNNs to form convolutional features containing both static spatial and dynamic temporal information. As the extracted dynamic features contain only low-level temporal information generated among image frames, we further used a modified recurrent network structure for high-level quality perception through a long time scale. In an attempt to simulate the temporal hysteresis effect of the human visual system, a weighted average evaluation model with adaptive weighting parameters was developed to generate the final scores. In order to verify the validity and generalization performance of the model, we conducted experimental validation on four public video quality datasets (CVD2014, KoNViD-1k, LIVE-Qualcomm, and LIVE-VQC) using SROCC and PLCC as metrics. The results reveal the superiority of the proposed method over the current state-of-the-art methods, which demonstrates that it is perfectly feasible to fuse multidimensional information followed by reasonable temporal sequencing in NR-VQA.

Current 3D convolution is computationally intensive and time-consuming, making it challenging to train evaluation networks end-to-end, and thus hindering further improvements in network performance. In the future, it will be important to find a more lightweight multidimensional feature extraction module for video quality awareness to enable end-to-end network training.

## Figures and Tables

**Table 1:** TMB architectures considered in our experiments. Each FC layer of the TMB block place the feature into GRU after shrinking. The left and right side of the arrow represent the feature size before and after shrinking, respectively. The output vector of width 64 is finally fed to a FC layer that outputs a quality score in the regression block.

Block Name	Output Size	GRU	FC
Block 1	384	256→128	5120→256
Block 2	160	128→32	384→128
Block 3	64	32→32	160→32
Regression	1	−	64→1

**Table 2:** Comparison of four video quality benchmark datasets: CVD2014, KoNViD-1K, LIVE-Qualcomm and LIVE-VQC.

	CVD2014 [59]	KoNViD-1k [60]	LIVE-Qualcomm [61]	LIVE-VQC [62]
Number of Videos	234	1200	208	585
Video Resolutions	640 × 480, 1280 × 720	960 × 540	1920 × 1080	320 × 240–1920 × 1080
Video Length	11–28 s	8 s	15 s	10 s
Video Frame Rate	9–30 frames/s	23–29 frames/s	30 frames/s	19–30 frames/s
Number of Devices	78	>164	8	101
Format	AVI	MP4	YUV	MP4
Distortion Type	Camera capture	Diverse distortions	Camera capture	Diverse distortions
Test Environment	Laboratory	Crowdsourcing	Laboratory	Crowdsourcing
Number of Test Subjects	210	642	39	4776
Rating Scale	[−6.50, 93.38]	[1.22, 4.64]	[16.5621, 73.6428]	[6.2237, 94.2865]

**Table 3:** Performance of the cross-dataset quality evaluation. The largest values marked in bold stand for the best results. The results of FRIQUEE, VBLIINDS, CRONIA, and TLVQM reported in [2] are shown here for reference.

**Training Database**	**CVD2014**			**KoNViD-1k**		
**Testing Database**	**KoNViD-1k**	**LIVE-Qualcomm**	**LIVE-VQC**	**CVD2014**	**LIVE-Qualcomm**	**LIVE-VQC**
TLVQM [2]	0.54	0.38	-	0.34	0.47	-
FRIQUEE [70]	0.49	0.09	-	0.62	0.38	-
VBLIINDS [37]	0.30	0.06	-	0.16	0.49	-
MDTVSFA [65]	0.6051	**0.3919**	0.4950	0.6474	**0.6732**	0.7160
Ours	**0.650**	0.389	**0.624**	**0.712**	0.616	**0.728**
**Training Database**	**LIVE-Qualcomm**			**LIVE-VQC**		
**Testing Database**	**CVD2014**	**KoNViD-1k**	**LIVE-VQC**	**CVD2014**	**KoNViD-1k**	**LIVE-Qualcomm**
CORNIA [71]	0.36	0.38	-	-	-	-
MDTVSFA [65]	0.5879	0.6128	0.6214	0.4819	0.7059	0.6550
Ours	**0.615**	**0.688**	**0.716**	**0.603**	**0.694**	**0.665**

**Table 4:** General performance comparison with state-of-the-art on CVD2014, KoNViD-1k, and LIVE-Qualcomm.

	BRISQUE [26]	VBLIINDS [37]	VIIDEO [72]	TLVQM [2]	MDTVSFA [65]	Ours
PLCC	0.603	0.613	0.235	0.77	0.792	0.799
SROCC	0.661	0.663	0.237	0.77	0.786	0.799

**Table 5:** Overall performance evaluation on four VQA databases. Results not reported are replaced with the “-” symbol.

Methods	CVD2014		KoNViD-1K		LIVE-Qualcomm		LIVE-VQC	
	SROCC	PLCC	SROCC	PLCC	SROCC	PLCC	SROCC	PLCC
BRISQUE [26]	0.709	0.715	0.654	0.626	0.504	0.516	0.569	0.587
CORNIA [73]	0.614	0.618	0.610	0.608	0.460	0.494	0.595	0.593
VBLIINDS [37]	0.746	0.753	0.695	0.658	0.566	0.568	0.702	0.712
VIIDEO [72]	0.023	−0.025	0.298	0.303	0.127	−0.001	0.150	0.245
TLVQM [2]	0.83	0.85	0.78	0.77	0.78	0.81	-	-
MDTVSFA [65]	0.831	0.841	0.781	0.786	0.802	0.822	0.738	0.772
VIDEVAL [64]	-	-	0.783	0.780	-	-	0.752	0.751
3D-CNN+LSTM [56]	-	-	0.800	0.808	0.687	0.792	-	-
Temporal pooling [57]	-	-	0.676 (+0.173)	0.717 (+0.136)	-	-	-	-
Ours	0.847	0.849	0.789	0.788	0.807	0.823	0.776	0.789

**Table 6:** Comparison of different input features and their fusions (Res50* refers to the model finetuned in the IQA task).

Feature	SROCC				PLCC			
	CVD2014	KoNViD-1k	LIVE-Qualcomm	LIVE-VQC	CVD2014	KoNViD-1k	LIVE-Qualcomm	LIVE-VQC
Res50	0.842	0.768	0.782	0.737	0.841	0.772	0.811	0.770
Res50*	0.842	0.768	0.787	0.768	0.843	0.766	0.809	0.791
R(2+1)D	0.803	0.721	0.743	0.693	0.801	0.714	0.779	0.709
R3D	0.789	0.678	0.745	0.720	0.804	0.671	0.766	0.736
R3D+R(2+1)D	0.802	0.734	0.805	0.732	0.818	0.732	0.826	0.737
Res50+R3D	0.836	0.776	0.796	0.772	0.844	0.778	0.807	0.791
Res50+R(2+1)D	0.839	0.788	0.784	0.772	0.848	0.784	0.802	0.789
Ours	0.847	0.789	0.807	0.776	0.849	0.788	0.823	0.789

**Table 7:** Performance of sum fusion compared with concatenation fusion. The decline in average performance is shown in parentheses.

	CVD2014	KoNViD-1K	LIVE-Qualcomm	LIVE-VQC
SROCC	0.817 (−0.03)	0.744 (−0.045)	0.788 (−0.019)	0.728 (−0.048)
PLCC	0.819 (−0.03)	0.740 (−0.048)	0.803 (−0.020)	0.740 (−0.049)

**Table 8:** Comparison of different long-term dependency models.

	Transformer		LSTM		GRU	
	SROCC	PLCC	SROCC	PLCC	SROCC	PLCC
CVD2014	0.798	0.734	0.841	0.847	0.847	0.849
KoNViD-1K	0.775	0.765	0.786	0.784	0.789	0.788
LIVE-Qualcomm	0.797	0.746	0.798	0.815	0.807	0.823
LIVE-VQC	0.746	0.724	0.778	0.791	0.776	0.789

## Data Availability

The used datasets were obtained from publically open source datasets from: 1. CVD2014: http://www.helsinki.fi/psychology/groups/visualcognition/ or https://zenodo.org/record/2646315#.YI-a4bUzaUl (alternative).( accessed on 11 January 2021) 2. KoNViD-1k: http://database.mmsp-kn.de/konvid-1k-database.html.( accessed on 4 January 2021) 3. LIVE- Qualcomm: https://live.ece.utexas.edu/research/incaptureDatabase/index.html.( accessed on 3 January 2021) 4. LIVE-VQC: https://live.ece.utexas.edu/research/LIVEVQC/index.html (accessed on 4 January 2021).
